# Differences in Textile Metal Threads in Croatia Through the Centuries

**DOI:** 10.3390/ma19112290

**Published:** 2026-05-28

**Authors:** Kristina Šimić, Ozana Martinčić, Damir Doračić, Tanja Pušić

**Affiliations:** 1 Faculty of Textile Technology, University of Zagreb, 10000 Zagreb, Croatia; tanja.pusic@ttf.unizg.hr; 2 Miroslav Krleža Lexicographic Institute, 10000 Zagreb, Croatia; ozana.martincic@lzmk.hr; 3 Archaeological Museum in Zagreb, 10000 Zagreb, Croatia; ddoracic@amz.hr

**Keywords:** textile metal threads, Croatia, archaeological textiles, historical textiles, microscopy, SEM-EDX

## Abstract

By studying and analysing a great number of textile metal threads from different time periods in Croatia, from archaeological sites to preserved specimens from various museum collections, change can be seen through the centuries. Metal threads decorate textiles not only for aesthetic purposes, but also to display wealth, assert authority, and command respect. Primarily, such decorated clothing was worn for liturgical purposes, ceremonial folk customs, or to mark high military ranks. Through the characteristics of these threads, one can see the change in the customs and lives of the people who used them in different ways and for different purposes. The shine and luxury that the first gold metal threads have can be achieved with lower quality threads like silver, copper, and their alloys. As the quality of metal textile threads decreased, they became cheaper and more applicable in everyday life. The use of metal textile threads on clothing increased, and through the century, they became a clothing part for all people, not just the privileged. Analyses of metal threads were performed with scanning electron microscopy with an energy-dispersive X-ray detector (SEM-EDX) due to its sensitivity and suitability for the observation of metal threads from various textiles. The type of textile cores from the combined textile metal threads was determined through a laboratory optical microscope. Differences have been observed between archaeological and historical textile metal threads in terms of physical properties, as well as textile and metal composition. Archaeological samples are combined textile metal threads that have a metal component of gilded silver and a textile component of silk. While more recent historical samples have different types of metal threads, from individual threads of lamellae and wires to combined textile metal threads. Most samples have cotton as a textile component, while copper, alone or in alloys, predominates in the metal threads.

## 1. Introduction

The earliest dated findings of metal threads intended for textile decoration in Croatia are the specimens from Knin site Greblje. They consist of 32 fragments of gold lamellae found in a female grave dated to the 6th century. Their location in the skull area suggests a possible function as a decoration on a headband. The width of the lamellae is between 0.75 and 1.25 mm, and they are shaped in a geometric V motif. The organic residue of the textile that served as the substrate has not been preserved due to deterioration from the age of the material [1,2].

In previous research, historical metal textile threads from the 17th to the 20th century in Croatia were analysed, 73 different threads from liturgical vestments and 83 threads from folk costumes. The SEM-EDX method was used for this type of sample as the most suitable for analysis, and a cross-section analysis was also performed [3]. The analysis of archaeological metal textile threads from the 8–9th to the 16th centuries was expanded, and a comparison was made according to the centuries and the directions in which the threads developed. Both archaeological and historical metal threads are highly valuable materials that provide insights into the past and the way of life for the people who lived at that time. By comparing them, it can be seen how they changed over the centuries and how the lives of the people who used them changed [4].

Archaeological textiles are usually found during archaeological excavations in specific, extreme locations such as graves, and are most often already in very poor and fragmented condition. Such valuable archaeological textiles are difficult to find due to decay and contamination, so the available samples are very few and their dimensions are extremely small. The analysis of such small and contaminated samples is very demanding, while the poor condition of the samples further complicates the process [5]. Historical textiles refer to items preserved in museums, churches, or collections that have been continuously maintained and are generally better preserved, often in complete form, which makes it difficult to obtain samples for analysis [6]. Archaeological specimens found in mediaeval cemeteries in Croatia are made in combination with threads of precious metals spirally twisted around an organic core. Only two finds from a grave near Glina date back to the early Middle Ages (late 8th–9th century) [7]. The cemetery around the pre-Romanesque church of St Spas is at the very source of the Cetina River. The cemetery, together with the church, occupied a surface of 1000 m^2^ and consisted of 150 graves [8]. At the mediaeval site of Crikvina, not far from Smiljan in Lika, two Romanesque churches were discovered: an older one, built in the second half of the 13th century, and a younger one, built soon after. Next to the churches was a multilayered cemetery of the inhabitants, and traces of textiles were found in one of the graves [9]. At the location of Štale in Bribir near Novi Vinodolski there is a cemetery where burials took place from the 11th to the 15th century. In one grave, fragments of a textile strip were found under a skull in a decomposed state, made of silver metal threads wrapped around organic yarn [10]. A systematic archaeological investigation of a mediaeval site at Crkvina near Gospić, which has a Romanesque church and cemetery, has been conducted. According to the findings, the site dates from the 12th to the 15th century, and two woven textiles were found [11,12]. The finds from Udbina, made of precious metal threads wrapped around an organic silk core, were mostly found in masonry tombs, suggesting that they belonged to the most distinguished social classes. These valuable finds are stored in the Archaeological Museum in Zadar [13].

SEM-EDX analysis is the most suitable and usual for metal thread samples; it reveals morphology with BSE images and EDX spectra detect the chemical composition. Also, SEM-EDX analysis can detect the type and amount of corrosion products developed on the surface of metal threads [14,15,16]. The presence of copper has a particular contribution to the preservation of organic fibres, since this element has pronounced antimicrobial properties and acts as a preservative in a certain way [17].

This research is focused on archaeological samples from the 8–9th centuries to the 16th century, as well as historical samples from the 17th to the 20th century. Studying and comparing archaeological and historical textile metal threads not only chronologically traces the development and change in textile metal threads but also of costumes and of life in Croatia through the centuries. The results of textile metal threads analyses were compared according to appearance: thread type, twist direction and width, as well as according to composition of the metal thread and the textile from the core.

## 2. Materials and Methods

Observed materials formed two groups of samples: archaeological findings from the sites and historical samples obtained from various museums. The analysed luxury textiles consist of 34 archaeological samples (Table 1), of which 9 sample analyses from Udbina are being published for the first time, and 156 historical samples, from liturgical vestments to folk costumes (Table 2).

**Figure 1:**
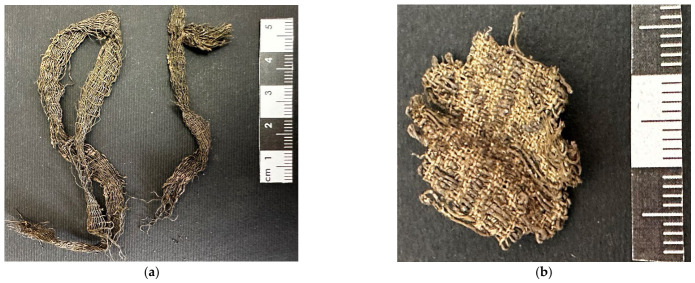
Archaeological samples, gilded silver from Udbina (**a**) from 13 to 14th century; (**b**) from 15 to 16th century.

Figure 1 shows two archaeological samples from Udbina but with different dates. The older sample has less visible golden shine due to greater decay from corrosion products than the newer sample.

**Figure 2:**
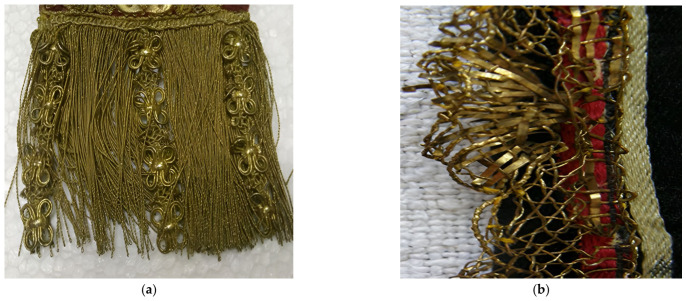
Historical samples (**a**) fringe from the Treasury of the Zagreb Cathedral Mitre from 17 to 18th century (liturgical vestment), fringe length 10 cm; (**b**) detail of the lace from the Museum of Slavonia Osijek apron from 19 to 20th century (folk costume), lace width 4 cm.

Figure 2 shows details of two historical samples, one from a liturgical vestment Mitre and the other from a folk costume apron. Fringe from Mitre is a combined textile metal thread, the gilded silver sample is from the Treasury of the Zagreb Cathedral and dates from the 17th to 18th century. The lace on the apron from the Museum of Slavonia in Osijek contains two metal threads: a single metal thread, a lamella, and a combined textile metal thread. It dates from the 19th to 20th century, and copper predominates in the composition of the metal thread.

Metal threads were characterised using a Scanning Electron Microscope (SEM) coupled with Energy-Dispersive X-ray Spectroscopy (EDX), equipped with a silicon drift detector (SDD) featuring a thin Si_3_N_4_ window, COXEM Co., Ltd., Daejeon, Republic of Korea. The detector resolution was 128 eV at the Mn Kα line. Analyses were performed at an accelerating voltage of 20 kV and a working distance of approximately 13 mm. Instrument calibration was verified using SRM^®^1107 (UNS C46400 naval brass, National Institute of Standards and Technology, Gaithersburg, MD, USA) which demonstrated an accuracy within ±12% of the certified standard values; however, no additional standards were employed during the analyses. It should be noted, though, that standard reference materials are typically homogeneous, not corroded, flat and polished—conditions required for accurate quantitative analysis but almost impossible to achieve when examining archaeological materials using surface-based, not destructive, analytical techniques, because of factors previously discussed in the literature [18,19,20]. Consequently, relative errors for elements detected in archaeological samples are generally higher, particularly for elements present at lower concentrations [18]. The primary objective of the analyses was to determine the general composition of the metal threads and identify possible traces of plating, rather than to obtain precise quantitative data or detect minor and trace elements. Therefore, despite the well-known limitations of analysis without standard [21] and uncertainties associated with the heterogeneous nature of archaeological metal alloys [20], qualitative and without standard semi-quantitative approaches were considered sufficient for the purposes of this investigation. Relative errors calculated by the instrument software during the analyses ranged from 5 to 16% for elements present at concentrations above 5 wt.% and from 25 to 80% for elements present at concentrations below 5 wt.%. Micrographs of the selected areas were acquired in backscattered electron (BSE) mode.

Detection of fibres from archaeological samples isolated from combined metal textile thread samples was obtained using a Kern OBE 134 laboratory optical microscope, KERN & SOHN GmbH, Balingen, Germany, magnification 100×.

## 3. Results

Textile metal thread samples were examined and structural analysis was performed. First, the type of metal thread and the direction of the thread twist were determined by textile conventional decomposition. An optical microscope was used to analyse the textile core from the combined textile metal thread. The width of the metal thread was measured on the SEM-BSE image.

**Figure 3:**
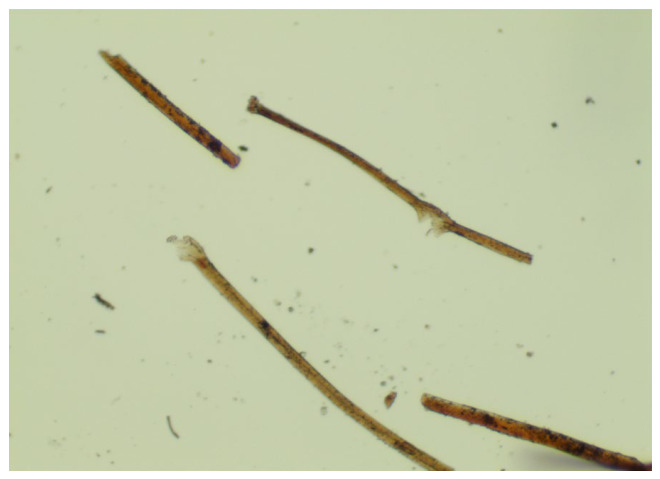
Optical microscope image of archaeological sample of textile core (silk) from Udbina from 13 to 14th century, 100× magnification.

**Figure 4:**
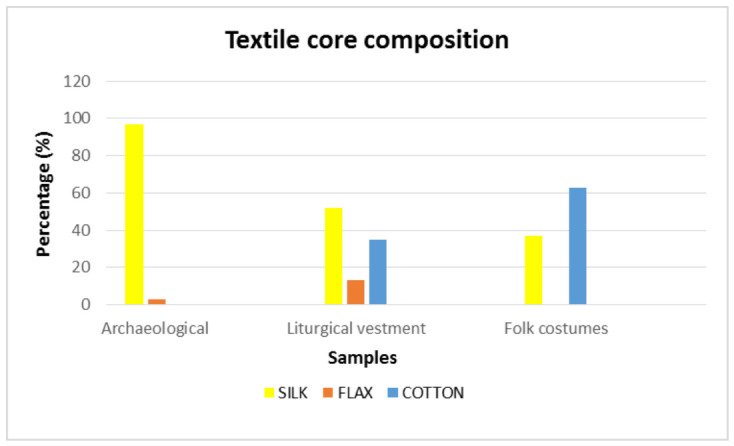
Graphical representation of the textile core composition in combined textile metal threads from archaeological and historical samples (liturgical vestments and folk costumes).

All archaeological samples specified in Table 1 have metal threads wrapped around a textile core in the S twist direction, as can be seen from samples from Udbina. While historical ones from Table 2 have three different threads, wires, lamellae, and combined textile metal threads. Combined textile metal threads have threads with both twist directions S and Z. Analysis of textile cores from combined textile metal threads indicates the presence of silk, Figure 3 and only one sample of flax in threads from the 8th–9th century to the 16th century. While younger historical textile threads indicate the presence of cotton, which increases in samples from folk costumes as the 20th century approaches, Table 3 and Figure 4.

**Figure 5:**
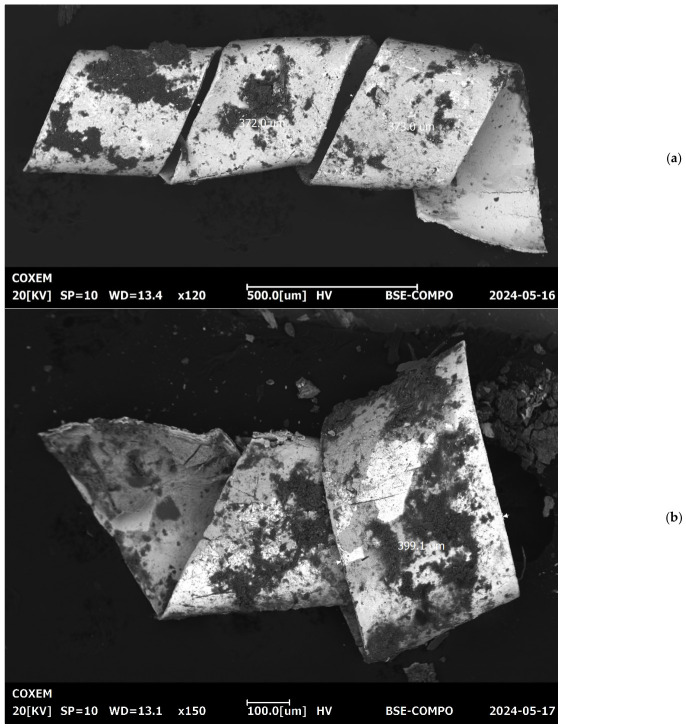
SEM-BSE images of gilded silver metal thread from Udbina showing measured thread widths: (**a**) 13–14th century; (**b**) 15–16th century.

The width of metal threads in archaeological combined metal textile threads ranges from 130 to 500 µm, Figure 5. The range of metal thread widths in combined threads from the 17th to 20th centuries is from 200 to 400 µm, while the width of the individual metal threads, lamellas, and wires differs. The width of lamellas is larger and ranges from 500 to 700 µm, but the wire diameter is from 70 to 100 µm [22]. A significant difference is observed in the size of the lamellae used individually from historical samples of the smaller lamellae used in combined metal textile threads.

Observed differences in the preservation of the golden shine can be explained by variations in the thickness of the gilding layer and the condition of the silver, Figure 5. In some finds, the thickness of the gilding is greater, which made it possible to preserve the intensity of the golden shine for a longer period. In other specimens, due to the thinner application of gilding and long-term abrasive action, in some areas, there was a complete loss of gilding. In addition, gold-plated silver threads do not retain their characteristic golden shine due to corrosion processes on silver that cause destabilisation and degradation of the gold layer. The analysed findings confirm that in the early period, metal threads were mainly made of gold. While later, for the sake of more economic and simpler processing, the use of silver, especially gold-plated silver, but also other less precious metals, was introduced as a more functional and affordable alternative, Table 4. As shown in a 2016 paper, copper and silver are the most commonly used historical metal threads from Croatian textiles [23].

**Figure 6:**
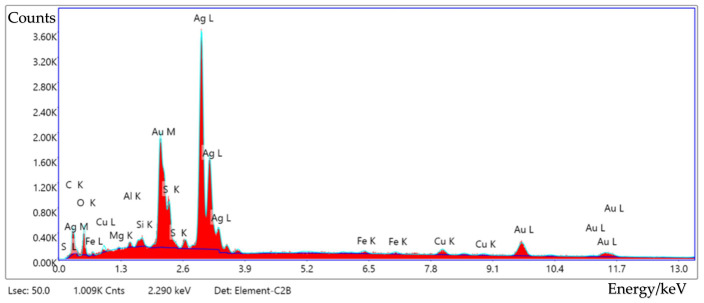
SEM-EDX analysis of gilded silver thread from Udbina (13–14th century): outer side of the metal thread.

**Figure 7:**
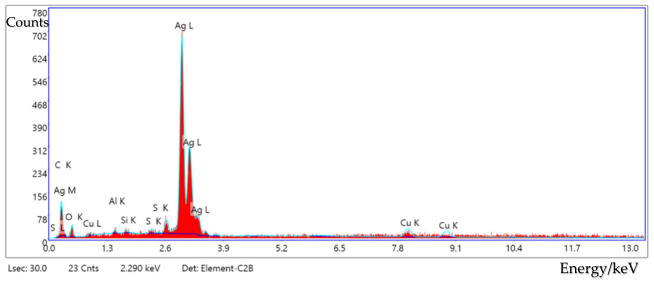
SEM-EDX analysis of gilded silver thread from Udbina (13–14th century): inner side of the metal thread.

**Figure 8:**
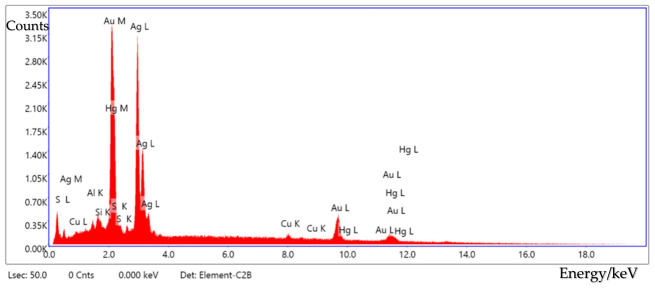
SEM-EDX analysis of gilded silver thread from Udbina (15–16th century): outer side of the metal thread.

**Figure 9:**
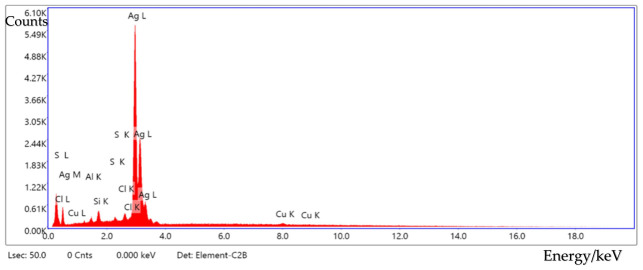
SEM-EDX analysis of gilded silver thread from Udbina (15–16th century): inner side of the metal thread.

SEM-EDS analysis was performed on both the outer surface and inner surface of the metal threads. The latter is typically obscured as it is wrapped around the textile core. Elemental characterisation of the threads showed that among the nine metal thread samples from Udbina, eight were identified as gilded silver and one as pure silver. In the broader archaeological assemblage, 22 samples were gilded silver, two were pure silver, and one was pure gold. On gilded silver samples, the gold coating was applied only to the outer, visible surface, Figure 6, Figure 7, Figure 8 and Figure 9. The oldest specimen, composed of pure gold, dates to the 8th–9th century, while the silver specimen dates from the 11th–15th century. Analysis of threads from the 17th to 20th centuries revealed a shift toward diverse compositions [3], with copper-based alloys becoming dominant (Table 4).

**Figure 10:**
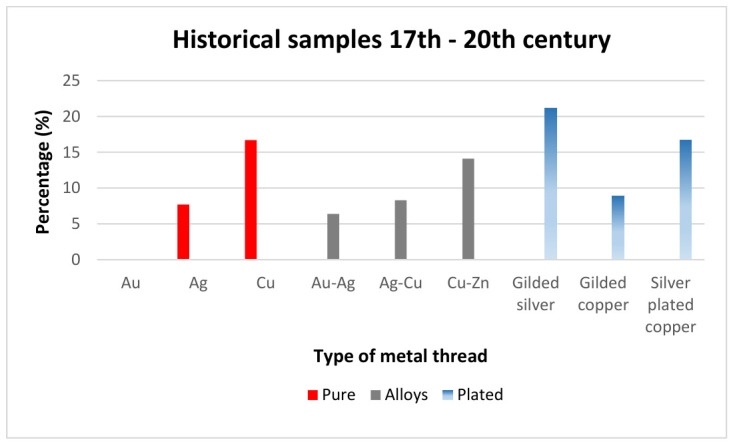
Graphical representation of different metal thread types in historical samples.

Unlike archaeological samples whose metal threads are mostly made of gilded silver, historical samples have very different types of metal threads, Table 4. Figure 10 shows in detail the percentage of all metal thread types in historical samples. Copper is present in all types of metal threads, pure metal threads, alloys and layered metal threads, and also in the highest percentage.

## 4. Discussion

Considering the state of the archaeological samples, SEM-EDX analysis provided critical data that contradicted initial visual inspections. While some threads exhibited an intense golden lustre suggestive of pure gold and others appeared dark grey, indicating tarnished silver, chemical analysis revealed their true multilayered structure. Most archaeological samples were gilded silver, with the gilding restricted to the visible outer surface.

The absence of mercury in the gilding layer of most of the silver threads (Figure 6) strongly suggests the use of diffusion bonding. In this process, gold foil and silver are joined at elevated temperatures (approx 300 °C), creating a thin interdiffusion layer that ensures a robust metallurgical bond [24]. Although mercury is present in small but detectable amounts (˂1.5%) in some samples (Figure 8), which could suggest fire gilding, the levels detected are an order of magnitude lower than the minimum threshold for amalgam-based techniques (5%) and therefore most likely indicate contamination originating from the goldsmith’s workshop [25]. Copper found in trace amounts was likely an intentional addition to the silver base to enhance mechanical strength and durability during the manufacturing of wires or strips [26]. Despite better preservation, threads from the 17th–20th centuries show a notable decline in material quality, with copper being the dominant alloying metal. Notably, pure gold threads are completely absent. The textile cores also show a chronological shift: archaeological metal threads are predominantly wound around silk or occasionally flax, whereas cotton becomes the dominant core material in 17th–20th century samples, often in combination with copper-based metal threads. Finally, a distinct technological marker was observed in the twist direction; archaeological samples exclusively exhibit an S-twist, while the Z-twist appears in threads from the later 17th to 20th century period.

## 5. Conclusions

Metal textile threads dating from the 8th–9th to the 20th century represent highly valuable historical material. Archaeological metal threads are relatively rare and often survive in limited amounts. These samples are extremely difficult to date precisely, as their poor state of preservation, characterised by corrosion and contamination, complicates analysis.

In archaeological samples, the metal component is typically composed of precious metals, primarily gold and silver, while the textile core is most often made of silk.

In contrast, metal threads from the 17th to the 20th century exhibit a markedly different composition. The proportion of gold and silver threads decreases significantly, while copper-alloy threads predominate. The textile cores of these later samples are predominantly made of cotton, whereas silk appears mainly in gold or silver-gilt threads. Overall, a clear decline in the quality of both metal and textile components can be observed over time. Metal threads become less costly and easier to produce, leading to their wider availability and use among broader segments of society. At the same time, the threads tend to become wider and are increasingly employed in the decoration of clothing.

## Figures and Tables

**Table 1:** Archaeological samples.

Location	Number of Samples	Dating
Grave near Glina	2	late 8th–9th centuries
Cemetery in Udbina	9 (new)	13th–16th centuries
Cemetery in Cetina	18	13th–15th centuries
Grave in hinterland of Novi Vinodolski	1	14th–15th centuries
Grave in Gospić	2	12th–15th centuries
Grave near Smiljan	1	13th–15th centuries
Grave near Novi Vinodolski	1	11th–15th centuries

**Table 2:** Historical samples.

Preservation Place	Group	Number of Samples	Dating
Treasury of the Zagreb Cathedral	liturgical vestments	14	17th–18th centuries
Varaždin City Museum	liturgical vestments	18	17th–19th centuries
Prilišće History Museum	liturgical vestments	16	18th–20th centuries
Novigrad History Museum, near Zadar	liturgical vestments	16	19th centuries
Museum of Slavonia Osijek	liturgical vestments	9	18th–20th centuries
Ethnographic Museum Zagreb	folk costumes	26	19th–20th centuries
Museum of Cetina region	folk costumes	8	18th century
Ethnographic Museum Split	folk costumes	16	17th–19th centuries
Ethnographic Museum Dubrovnik	folk costumes	18	19th–20th centuries
Museum of Slavonia Osijek	folk costumes	15	19th–20th centuries

**Table 3:** The main differences between older archaeological and more recent historical metal threads.

Sample	Type of Metal Thread	Thread Twist Direction	Type of Textile Core
From 8–9th to 16th century	– Combined metal textile threads	S	– Silk – Flax
From 17th to 20th century	– Combined metal textile threads – Lamellas – Wires	S and Z	– Cotton – Silk – Flax

**Table 4:** Different metal threads from archaeological (8–9th to 16th century) to historical (17th to 20th century).

Metal Threads	From 8–9th to 16th Century	From 17th to 20th Century
Gold	1	-
Silver	3	12
Gilded silver	30	33
Silver-plated copper	-	26
Copper	-	26
Gilded copper	-	14
Gold–silver alloy	-	10
Silver–copper alloy	-	13
Copper–zinc alloy	-	22

## Data Availability

The original contributions presented in this study are included in the article. Further inquiries can be directed to the corresponding author.
